# Involvement of EphB1 Receptors Signalling in Models of Inflammatory and Neuropathic Pain

**DOI:** 10.1371/journal.pone.0053673

**Published:** 2013-01-16

**Authors:** Vincent Cibert-Goton, Guanglu Yuan, Anna Battaglia, Sarah Fredriksson, Mark Henkemeyer, Thomas Sears, Isabella Gavazzi

**Affiliations:** 1 Wolfson Centre for Age-Related Diseases, The Wolfson Wing, King’s College London, Guy’s Campus, London, United Kingdom; 2 Department of Developmental Biology, Kent Waldrep Center for Basic Research on Nerve Growth and Regeneration, University of Texas Southwestern Medical Center, Dallas, Texas, United States of America; University of Würzburg, Germany

## Abstract

EphB receptors tyrosine kinases and ephrinB ligands were first identified as guidance molecules involved in the establishment of topographical mapping and connectivity in the nervous system during development. Later in development and into adulthood their primary role would switch from guidance to activity-dependent modulation of synaptic efficacy. In sensory systems, they play a role in both the onset of inflammatory and neuropathic pain, and in the establishment of central sensitisation, an NMDA-mediated form of synaptic plasticity thought to underlie most forms of chronic pain. We studied wild type and EphB1 knockout mice in a range of inflammatory and neuropathic pain models to determine 1), whether EphB1 expression is necessary for the onset and/or maintenance of persistent pain, regardless of origin; 2), whether in these models cellular and molecular changes, e.g. phosphorylation of the NR2B subunit of the NMDA receptor, increased c-fos expression or microglial activation, associated with the onset of pain, are affected by the lack of functional EphB1 receptors. Differences in phenotype were examined behaviourally, anatomically, biochemically and electrophysiologically. Our results establish firstly, that functional EphB1 receptors are not essential for the development of normal nociception, thermal or mechanical sensitivity. Secondly, they demonstrate a widespread involvement of EphB1 receptors in chronic pain. NR2B phosphorylation, c-fos expression and microglial activation are all reduced in EphB1 knockout mice. This last finding is intriguing, since microglial activation is supposedly triggered directly by primary afferents, therefore it was not expected to be affected. Interestingly, in some models of long-term pain (days), mechanical and thermal hyperalgesia develop both in wild type and EphB1 knockout mice, but recovery is faster in the latter, indicating that in particular models these receptors are required for the maintenance, rather than the onset of, thermal and mechanical hypersensitivity. This potentially makes them an attractive target for analgesic strategies.

## Introduction

The discovery that members of the EphB subfamily of receptor tyrosine kinases are involved in the formation of glutamatergic synapses [1] led rapidly to the finding that these molecules are not only required for axonal guidance and neuronal circuit assembly during development, but are also involved in synaptic plasticity during development and into adulthood. The majority of studies on adult plasticity have focused on EphB-ephrinB interactions in the onset and/or maintenance of long-term potentiation (LTP) in different areas of the hippocampus, and in the striatum [2], [3], where significant advances in establishing mechanisms and functional significance of Eph and ephrin involvement in synaptic plasticity have been made using genetically modified mice [3]. The use of transgenic mice proved necessary to study these molecules for a variety of reasons, primarily linked to the binding promiscuity of the different EphB receptors, and the bidirectional signalling activated by EphB-ephrinB. There are 5 EphB receptors in mammals (EphB1-4 and EphB6), which bind promiscuously with 3 transmembrane ligands (ephrinB1-B3)(see e.g. [4]–[6]). Despite being conventionally called receptor and ligand, both EphBs and ephrinBs activate intracellular signalling cascades (forward and reverse signalling). The only tools currently available to interfere with EphB-ephrinB interaction, EphB-Fc or ephrinB-Fc chimeras, are not specific for a given receptor or ligand, and, whilst blocking the activation of EphB receptors or ephrinB respectively, can also bind to and activate ephrinBs or EphB receptors. The results obtained in rats and wild type mice are therefore unavoidably ambiguous, and studies in genetically modified animals are currently essential to clarify mechanisms.

Synaptic plasticity in which EphB-ephrinB interactions are involved has also been revealed at spinal cord level using models of chronic pain, thus providing an alternative model for the study of the link between molecular mechanisms and behavioural outcomes (see e.g. [7]–[10]).

In many models of chronic pain, a fundamental mechanism underlying enhanced responsiveness or spontaneous pain is central sensitisation, a mechanism which shares some features with hippocampal LTP, implying enhanced transmission at synapses formed by peripheral sensory afferents onto dorsal horn neurons; to what extent LTP itself contributes to central sensitisation and chronic pain is still debated (see e.g.[11], [12], [12]–[15]). Most, if not all, forms of central sensitisation are thought to be NMDA – mediated, but, despite three decades of research, controversy remains as to the specific molecular mechanisms and the role of NMDA receptor subunits, and even on the extent of NMDA receptor involvement (see e.g. [16]–[19]). Furthermore, there is a substantial body of evidence supporting the hypothesis that the mechanisms underlying the onset of different forms of chronic pain (e.g. inflammatory and neuropathic) as well as thermal and mechanical hyperalgesia, differ, even if there are contradictory findings also in relation to this issue (see e.g. [20]–[23]. It is clearly of importance therefore a), to establish in full the molecular mechanisms involved in the process of central sensitisation; b) to determine to what extent the same mechanisms are common to different types of chronic pain. This is essential to identify therapeutic targets for the development of effective mechanism-based analgesics hopefully with greater efficacy and fewer side effects than those currently available. Clarifying the contribution of EphB receptors to the spinal mechanisms of chronic pain would help achieving these goals.

Previous work carried out *in vivo* on adult rats in our laboratory demonstrated a requirement for spinal EphB receptor activation in NMDA-dependent increases in synaptic efficacy, using two models of chronic pain (formalin and carrageenan tests, [7]), and later EphB receptors were shown to be involved in neuropathic pain and the physical dependence on morphine [9], [10], [24]. Whereas in the hippocampus LTP is mainly dependent on EphB2, we suggested that in the spinal cord the primary receptor involved in chronic pain would be EphB1 [7], an hypothesis strongly supported by subsequent studies on EphB1 knockout (EphB1 KO) mice, which reported a lack of thermal hyperalgesia in KO mice in a model of neuropathic pain [25] and showed that EphB1 is essential for the generation of LTP between C-fibres afferents and dorsal horn neurons [26]. These findings suggest that a better understanding of the role of EphB1 receptor in chronic pain could, potentially, not only contribute to clarify the mechanisms underlying central sensitisation in different pain models, but provide an alternative therapeutic target for the treatment of chronic pain of different aetiology.

The aim of the present work was to advance understanding of the role played by EphB1 receptors in the onset of persistent pain, using EphB1 KO mice. We hypothesised that, if NMDA-mediated central sensitisation is indeed responsible for the onset of thermal and mechanical hyperalgesia, as well as spontaneous pain, in models of tissue injury, inflammatory and neuropathic pain, then EphB1 KO mice should display blunted pain responses in all these models. Furthermore, since NR2B phosphorylation and c-fos upregulation are thought to be downstream of EphB receptors activation ([27]; see also [7], [8]), we hypothesised that we would observe a reduction in NR2B phosphorylation and c-fos expression in KO mice in models of inflammatory pain, as we had previously shown to occur in rats, if we blocked the interaction between EphB receptors and ephrinBs ligands in vivo. We also examined microglial activation in neuropathic pain. The mechanisms leading to microglial activation in neuropathic pain models are still incompletely understood, but they appear to involve direct activation by primary afferents, possibly via the release of ATP, followed by activation depending on release of fractalkine from neurons, cleaved by Cathepsin S of microglial origin [28]. None of these mechanisms should be affected by the absence of EphB1. Previous studies had not analysed normal sensory function in EphB1 KO mice; there are no findings available from studies on embryos suggesting a role for EphB1 in the development of primary sensory afferents, but we deemed nonetheless essential to ascertain anatomically, behaviourally, immunohistochemically and electrophysiologically whether EphB1 deficiency caused any major developmental alterations in the functional connectivity of the sensory system. We were able to demonstrate that functional EphB1 receptors are not essential for the development of normal nociception, thermal or mechanical sensitivity, whereas, in agreement with our hypothesis, they are required for the onset and/or maintenance of thermal and mechanical hyperalgesia and spontaneous pain behaviour in a variety of persistent pain models. Surprisingly, microglial activation following nerve injury was reduced in EphB1 KO mice. Of particular interest is the finding that in some models of long-term pain mechanical and thermal hyperalgesia develop both in wild type and EphB1 knockout mice, but recovery is faster in the latter. This implies that in these models EphB1 receptors are required for the maintenance, rather than the onset, of hyperalgesia, and may therefore be useful therapeutic targets for the development of new analgesics.

## Materials and Methods

### Ethical Approval

All experiments were undertaken in accordance with the United Kingdom Animals (scientific procedures) Act 1986 (Project Licence PPL 70/4774), and all efforts were made to minimize animal suffering, and to reduce the number of animals used, in accordance with the concept of “The Three Rs” (*replacement*, *reduction*, *refinement*).

### Animals

This study has been conducted on EphB1 null mutant mice. Generation of the mutation has already been described [29]. We used EphB1−/− mutants backcrossed for several generation in the CD1 background to avoid possible confounding effects of background genetic differences, and EphB1 lacz/lacz mutants (expressing beta-galactosidase as a marker for EphB1 expression), backcrossed for several generations in a CD1/129 background. Aged-matched wild type (WT) littermates were used for comparison. Male and females mice were housed up to five per cage on a standard 12 hours light/dark cycle, with water and food pellets available ad lib except during the testing period. Animals were aged 12 to 15 weeks at the time of testing.

### Animal Models

A number of models of acute and persistent pain were used, i.e. the formalin test (a model of tissue injury [30], the Carrageenan and Complete Freund’s Adjuvant (CFA) models of inflammation and two models of neuropathic pain, chronic constriction injury (CCI) and partial nerve ligation (PNL).

For the formalin test, 20 µl of formalin at a 5% concentration (40% stock solution considered as 100%) were injected subcutaneously into the plantar surface of the right hind paw, using insulin syringes with 0.33 mm needles.

For the Carrageenan model of inflammation, each animal received a 20 µl injection of carrageenan (2% or 20 mg/ml Carrageenan Lambda, Type IV, Sigma-Aldrich, Gillingham, Dorset, UK) into the right hind paw.

For the CFA model of inflammation, 20 µl of CFA (Sigma F-5881, Sigma-Aldrich) were injected into the plantar surface of the right hind paw of the mouse.

Chronic constriction injury (CCI or Bennett model) [31]: for all animals, anesthesia was induced and maintained with 3% isofluorane in 95% oxygen, 5% CO2 via a face-mask. The left common sciatic nerve was exposed at the mid-thigh level and proximal to the sciatic nerve’s trifurcation, and four ligatures with chromic catgut were tied around it with about 1 mm spacing between ligatures. Each ligature was tightened in order to reduce the diameter of the nerve and retard, but not interrupt the epineural circulation.

Partial sciatic nerve ligation (PNL or Seltzer model) [32]: animals were anaesthetised as above. An incision was made in the skin of the upper left leg and blunt scissors were used to part the muscle layers to access the sciatic nerve. A tight ligation of between ½-⅓ of the nerve was made using 6-0 mersilk suture (Ethicon, Gargrave, North Yorkshire, UK). The skin was closed using 4-0 mersilk sutures (Ethicon). In sham animals, the surgery was performed as described above, but the nerve was not ligated.

### Behavioural Testing

All experiments were done during the day portion of the circadian cycle only (06∶00–18∶00 h) and were performed blinded to all test paradigms.

The Rotarod test was used to measure the balance and the forced motor-coordination activity. We used a combination of both accelerating and staggered Rotarod procedures on an accelerating Rotarod (IITC Inc. Life Science, Woodland Hills, CA) with a 32 mm diameter rod. On the first day the mice were given a limited habituation of 5 min on a non rotating rod. Forty eight h later they were tested 3 times at a rotating speed of 5 rpm and the latency to fall was measured for up to 2 min, with a 30 min inter-trial interval back in their home cages, to limit the effects of physical exhaustion. Every time a mouse was tested it was placed on a non rotating rod, which was accelerated to a defined rotating speed (5, 10, 20 and 30 rpm) with a constant incremental slope. Any mice falling before the rod reached its full speed was given 2 more chances. In case of 3 consecutive failures the given score was 0. The test was performed on 4 different days, every 48 h.

The Hargreaves’ Plantar test (IITC Inc. Life Science) was used to examine thermal sensitivity. Each mouse was housed separately, in a randomly chosen compartment. A glass heating system assured a standardized temperature of 22°C at the contact zone between the plantar surface of the paw and the glass (baseline temperature of the skin). Before the test, mice were habituated to the experimental set-up for 2 h minimum on 2 consecutive days prior and on the day of experiment. During the test, a heat source was applied to the plantar surface of the hind paw, from underneath and through the glass floor. The area tested was the mid-plantar hind paw, in the area supplied by the tibial nerve, avoiding the footpads, and was located by positioning a light spot on the plantar surface of the paw. The stimulus and a timer to record the reaction time were activated simultaneously. As soon as the mouse removed its paw, the stimulus and timer were stopped.

**Figure 1:**
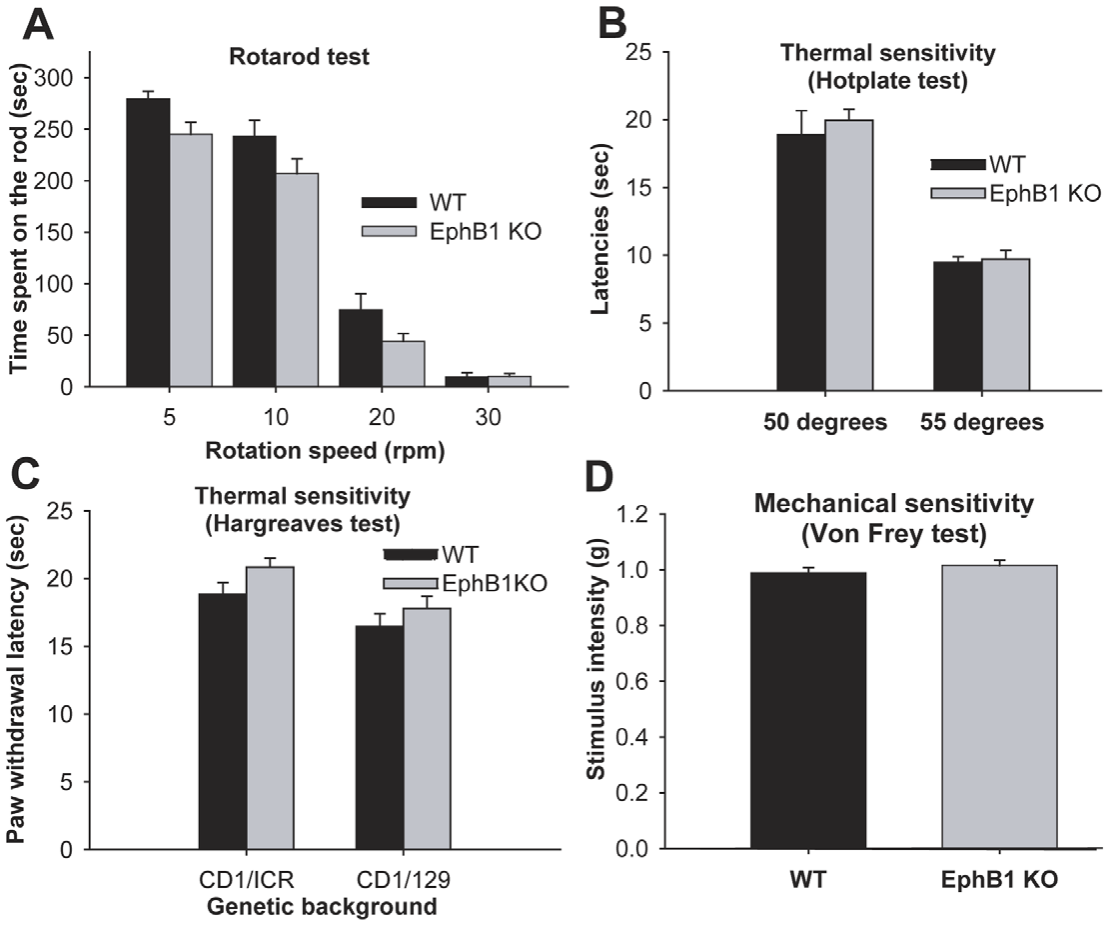
EphB1 KO mice have normal thermal and mechanical sensitivity. Bar charts illustrating the performance of EphB1 KO and WT mice in a range of behavioural tests designed to measure motor performance (rotarod, A) or acute thermal (hot plate and Hargreaves, B and C) or mechanical (von Frey, D) nociception. Behavioural analysis did not identify a significant difference between EphB1 KO and WT mice in any test. (t-test in A for 10 and 30 rpm, C and D; Mann-Whitney U-test in A for 5 and 20 rpm and in B) A) 5, 10, 20 rpm WT n = 19, EphB1 KO n = 29; 30 rpm WT n = 8, EphB1 KO n = 16; B) WT n = 16, EphB1 KO n = 25; C) CD1/ICR WT n = 24, EphB1 KO n = 17, CD1/129 WT n = 13, EphB1 KO n = 12. Bars represent mean +/− SEM.

After preliminary testing on control mice (WT CD1-ICR and CD1/129), the light intensity was set at 18% of maximum, in order to achieve a baseline of 10 to 20 seconds. The minimum resting period between each successive exposure was 5 min for alternate paws or 10 min for the same paw, to minimize the effects of repeated exposures. Two to 4 measurements were collected for each paw, depending on their reproducibility. For the data presented in figure 1, control WT and EphB1 mice were tested 3 times on 3 different days, with at least 2 days rest between test days. To examine the development of thermal hyperalgesia in inflammatory and neuropathic pain, animals were tested before treatment, and 90 and 150 min after carrageenan or saline injection [33], on day 1, 2, 5, 7, 9 and 14 after CFA or saline injection and at 3, 5, 7, 10, 14, and 21 days after PNL.

For the Hotplate test for thermal sensitivity, on the previous day and just before testing, mice were habituated for 10 minutes in the Hot Plate Plexiglas box (IITC Inc. Life Science). Mice from the same rearing box were habituated together, to minimize stress. After habituation, mice were placed individually on the hot plate, heated at either 50 or 55°C, and the latency to first sign of paw licking or escape jumping was recorded as an index of the pain threshold. The experiment was performed 3 times with 48 h between two measurements. A stimulus cut-off of 45 s and 1 min were used respectively when testing at 55 and 50°C to avoid any paw damage.

Mechanical sensitivity was tested using von Frey Hairs. To determine the baseline threshold for mechanical sensitivity for the mice used in our experiments (baseline mechanical sensitivity varies considerably in mice depending on genetic background), we used a series of 6 calibrated nylon monofilaments (Touch-Test Sensory Evaluator, Semmes-Weinstein Monofilaments) chosen with equal spacing (σ = 0.34 on average) on the log-dose scale, ranging from 0.04 g to 4.56 g. The mice were placed in individual plastic compartments on a stand with a metal mesh floor, and the von Frey hair applied perpendicularly to the plantar surface of the paw until it buckled slightly (minimum 20%), and maintained for 6 seconds. The next stimulus was presented as soon as any behavioural response due to the previous one was resolved. A positive response was recorded when the mouse sharply removed its paw, often followed by shaking and even licking [34]. Flinching immediately upon application or removal of the hair was considered a positive response. When assessing the baseline paw withdrawal threshold, each monofilament was applied five times on each paw at 5 min interval. The withdrawal threshold corresponded to the force in g at which the mouse withdrew the paw in minimum 5 out of 10 trials (combining the results of contra- and ipsi- lateral paws). Data for figure 1 were collected using this method. For all subsequent experiments (carrageenan and CFA i.pl. injection, neuropathic pain models) we adopted instead the up-and down method described by Chaplan et al. [34], which allows interpolation of the mechanical threshold (stimulus intensity at which the mouse would respond 50% of the times) with fewer applications of the von Frey filaments, and was therefore more suitable for these tests, inducing less stress in the animals. The up-and-down method requires prior knowledge of baseline mechanical threshold, so it could not have been applied in our initial experiments. Animals were tested before treatment, and 90 and 150 min after carrageenan or saline injection [33], on day 1, 2, 5, 7, 9 and 14 after CFA or saline injection, at 3, 5, 7, 10, 14, and 21 days after PNL Mechanical and at 7, 14 and 21 days after CCI.

For the Formalin test mice were housed in a square test box, maintaining a constant temperature of 22°C at the plantar surface of the paw [35]. Three out of the four sides of the box were mirrors, to simplify the observation and characterisation of the different behaviours recorded. Mice were habituated to the experimental environment for 30 min on two different days, and 15 min on the day of experiment. After formalin injection, mice were immediately transferred into the box and nocifensive responses (Lifting/Favouring; Licking/Biting) related to the injected hind paw, recorded in 5 min blocks for 1 h. A ‘painscore’ was then calculated according to the formula PS = (Time spent in full weight bearing position * 0)+(time spent lifting or favouring * 1)+(time spent licking or Biting * 2)).

### Immunohistochemistry

Mice were deeply anesthetized with pentobarbital (140 mg/kg, i.p.) and perfused transcardially with heparinised saline (0.9% NaCl) followed by 0.1M pH 7.4 phosphate buffer (PB) containing 4% paraformaldehyde (PFA). The left and right L4 and L5 lumbar dorsal root ganglia (DRGs) and/or lumbar spinal cords were then dissected and stored in 4% PFA overnight. The following day tissues were transferred in 20% sucrose in 0.1M PB, pH 7.4 for 24 h at at 4°C and then blocked in O.C.T. (Optimum Cutting Temperature) embedding matrix (BDH, Essex, UK) in liquid nitrogen and stored at −80°C. In all studies, 2–5 slides from each animal were used for the staining. 2 slides were randomly chosen as negative control for the staining process, 1 slide was coated only with primary antibody and the other with secondary antibody only. 15 µm thick transverse sections of L4 and L5 DRGs (perpendicular to the neuronal axis of the DRG) or 30 µm thick lumbar spinal cord section (L3-4, where afferents from the mouse hind limb preferentially terminate, see [36]) were cut on a Bright microtome 5040, mounted on slides (Superfrost Plus, BDH) sequentially, allowed to dry at room temperature for 30 min and then preserved in cryoprotectant (30% sucrose w/v, 30% ethylene glycol w/v in 0.01M phosphate buffer saline (PBS) at –20°C.

Lumbar DRG staining: three randomly chosen left or right DRGs from intact EphB1 KO mice and three randomly chosen left or right DRGs from intact WT mice were used, and slides were chosen from each animal so that 5 roughly equidistant sections taken from the entire length of the ganglion would be examined for each staining. The slides were then processed with conventional immunofluoscence techniques. To identify the proportion of peptidergic nociceptive neurons in the DRGs, the sections were stained for CGRP using primary antibodies rabbit anti-CGRP (Sigma c8198) at 1∶1300 and, to stain all neurons, with the general neuronal marker mouse anti-βIII-tubulin (Promega, Southampton, UK, 1 mg/ml) at 1∶1200 with secondary antibodies donkey anti-mouse CyIII (Jackson Immunochemicals, West Grove, PA, 1.3 mg/ml) and donkey anti-rabbit FITC (Jackson, 1.4 mg/ml). Non peptidergic nociceptive neurons were identified on the basis of their binding to isolectin B_4_ (IB_4_). IB_4_-biotin (Sigma, 0.5 mg/ml) was used in the concentration 1∶500 together with mouse βIII-tubulin (Promega, 1 mg/ml) at 1∶1200. As secondary antibodies, donkey anti-mouse FITC (Jackson, 1.4 mg/ml) at 1∶200 and anti-IB_4_ TRITC (Sigma, 2 mg/ml) at 1∶200 were used. Large, mainly proprio- or mechanoceptive, neurons were stained using mouse N52 antibody (Sigma) at 1∶400 and the general neuronal marker rabbit protein gene product (PGP) 9.5 (Abcam, Cambridge, UK) at 1∶400. The secondary antibodies used were donkey anti-rabbit FITC (Jackson, 1.5 mg/ml) at 1∶200 and donkey anti-mouse CyIII (Jackson, 1.3 mg/ml) at 1∶1000. The slides were then washed with 0.01M PBS (3×5 min) and covered with 22×50 mm Borosillicate glass cover slips (Calbiochem, San Diego, USA) using Flurosave™ Reagent (Sigma, St. Louis, MO, USA). All antibodies were diluted using 0.01M PBS with 0.2% Triton X (w/v) and 0.1% (w/v) of sodium azide.

Spinal cord sections: to examine the anatomical integrity of nociceptive afferent terminals in the dorsal horn of the spinal cord, the spinal cords from 3 WT and 3 EphB1 KO mice were dissected. 3 slides were then randomly chosen from each animal and double-stained for CGRP and IB4. Sections were stained for CGRP using as primary antibody a rabbit anti-CGRP (Sigma c8198) at 1∶1400 with secondary antibody Goat anti-rabbit Alexafluor 546 (Molecular Probes, Eugene, OR, USA). IB_4_-biotin (Sigma, 0.5 mg/ml) was used in the concentration 1∶500 and as secondary Extravidine FITC at 1∶500. To examine the effect of inflammation or tissue injury on the expression of c-fos, the spinal cords of 12 WT and 12 EphB1 KO mice were dissected out. Both WT and KO mice had been assigned randomly to one of 4 treatment groups: 1) saline i.pl injection 1 h prior to culling; 2) formalin i.pl. injection 1 h prior to culling; 3) saline i.pl. injection 3 h prior to culling; 4) carrageenan i.pl injection 3 h prior to culling; all injections were performed as described previously. Two slides were randomly chosen from each animal, then coated with a primary antibody rabbit anti-c-fos (1∶2500) (Invitrogen, Paisley, UK) and a secondary antibody Alexafluor 488 goat anti-rabbit IgG (1∶1000) (Molecular Probes). For assessment of microglial activation following PNL, 4–5 sections of spinal cords from WT and KO mice were randomly collected 3,7,14 and 21 days after lesion. Sham operated animals acted as controls. Slides were coated with Rabbit anti Iba1 (Wako, Japan) 1∶1000, followed by Goat anti rabbit IgG Alexa fluor 546 (Molecular Probes A11035) 1∶1000. All antibodies were diluted to working concentrations with 0.01M PBS with 1% (w/v) TritonX and 0.01% (w/v) sodium azide.

### Image Acquisition and Analysis

All images were acquired using a Zeiss fluorescence microscope (Axioplan 2 or Imager Z1) with a 20× objective with a Zeiss Axiocam HRm (Carl Zeiss, Goettingen, Germany), using Axiovision 40LE version 4.2 software (Carl Zeiss). All analysis was performed with the investigator blinded to genotype and treatment.

Lumbar DRGs: Two randomly chosen pictures were taken using a Zeiss Axioplan 2 microscope. All stained cells with visible nuclei were counted, first in sections stained with the general neuronal marker, then in sections stained with the subpopulation-specific marker. For each animal, 5 sections were counted for each staining, for a total of 15 sections per animal. A total of 43660 cells were counted for WT, and 47827 cells for EphB1 KO mice.

Spinal cords: to quantify nociceptive afferents in the dorsal horn, photomicrographs of sections stained for CGRP immunorectivity or IB4 binding were taken using a Zeiss Imager.Z1 microscope. The surface of the stained area was measured using the outlining application in Axiovision 40LE version 4.2 software (Carl Zeiss). Immunoreactive c-fos expressing cells were counted in Laminae I and II of the ipsilateral (injected) side of the lumbar spinal cord grey matter.

To study microglial activation, usually 5 (or a minimum of 4) sections from each animal were chosen at random. Four boxes measuring 10^4^ µm^2^ were placed onto areas of the lateral, central and medial dorsal horn. The mean number of Iba1 positive cells, morphologically identified as activated microglia, was determined within this area [37]. All spinal cord sections were examined using a Zeiss Axioplan 2 microscope.

### Western Blot and Immunoprecipitation

Animals were divided into saline, CFA, carrageenan and formalin treated groups for both genotypes. The mice were culled and hemisected lumbar spinal cord tissues were rapidly removed at 60 minutes after formalin injection, 3 hours after carrageenan and 14 days after CFA. Saline controls were taken at the same time as their treated counterpart. Tissues were frozen in liquid nitrogen and stored at −80°C until use.

Each sample, obtained pooling the tissues from two mice, was homogenized on ice using an Ultraturax (IKA) homogeniser in 300 µl of NP-40 lysis buffer (1% Nonidet P-40, 150 mM NaCl, 500 mM Tris-HCl pH8, 1 mM EDTA, 0.5% SDS, adding 25× Protease inhibitor cocktail (Complete Roche), 100 µl leupetin (10 µg/ml), 100 µl phosphatase inhibitor cocktail 2 (Sigma), 200 µl NaF (250 mM stock) mix and 100 µl (1 mM final) PMSF to each 10 ml of buffer). 2×300 µl of lysis buffer was added to wash the Ultraturax to collect all the homogenate. The Ultraturax was also cleaned with ethanol and ddH_2_O between each sample homogenization. The homogenates were placed in a carousel for 2 hours at 4°C before being centrifuged at 12000 rpm for 20 minutes at 4°C. The supernatants were collected and the protein concentrations were determined using a detergent-compatible assay (BCA-Pierce kit) with a bovine serum albumin (BSA) standard in a Dynex reader (Dynex Technologies, Inc. Chantilly, VA).

For immunoprecipitation, total lysates (1 mg) were incubated with 5 µl of goat anti-NR2B IgG (Santa Cruz Biotechnology, Santa Cruz, CA, 0.2 µg/µl) on a carousel overnight at 4°C, followed by incubation with 50 µl of Protein G- Sepharose slurry (1∶1, Santa Cruz Biotechnology) at 4°C for 2 hours in agitation and further centrifugation at 5000 rpm for 2 minutes. Supernatants were discarded and Sepharose beads were washed 3 times with 1 ml of lysis buffer; with vigorous vortex and brief spin at 5000 rpm after each wash. 40 µl of 2 times SDS sample buffer was added to each sample, then vortexed and heated at 95°C for 5 minutes, before 5 minutes centrifugation at 12000 rpm. The immunoprecipitation samples were then kept at −20°C until loaded.

For western blot analysis, proteins (1 mg) were resolved on 8% SDS-PAGE gels run in migration buffer (3 g Tris-base, 14.4 g glycine, 1 g 1% SDS for 1litre) for 90 minutes at 90V and loaded along with controls (using normal goat serum) and full range rainbow recombinant marker (Amersham Biosciences, Arlington Heights, IL). Transfer was run in buffer (100 ml migration buffer (without SDS), 200 ml methanol, 700 ml H_2_O for 1 litre) for an hour at 100V using nitrocellulose membranes (Amersham) and a Trans-Blot Transfer Cell system (Bio-Rad, Hercules, CA) to which an icebox was added to avoid overheating and damage to the membrane. The blots were blocked at room temperature for 1 hour on agitation with 4% BSA in TBS-T (2.42 g Tris base, 8 g NaCl, adjust PH = 7.6 with HCl, 0.5 ml Tween 20, adding H_2_O for 1 litre) and the membrane incubated with two primary antibodies: mouse a-p-Tyr (1∶2000 TBS-T; Santa Cruz Biotechnology) and rabbit anti-NR2B IgG (1∶1000 TBS-T; Upstate 1 µg/µl) in a polythene container overnight at 4°C. The membrane was then washed 3 times for 10mins in TBS-T before being incubated with the secondary antibodies, goat anti-mouse IR680 and donkey anti-rabbit IR800 (both 1∶15,000–20,000 in TBS-T) for 1 hour on agitation. Revelation was carried out using the Odyssey Lyca Scan.

### Electrophysiology

Mice were given an initial dose of 1.8 g/kg urethane i.p. and 15 min later, 0.4 g/kg for sustained anaesthesia. Rectal temperature was maintained at a mean of 36–38°C. After tracheotomy, mice were supported prone by clamps at T13 and L3/4. L1-2 laminae were removed, the dura opened, and the tissues covered by 4% saline-agar which was subsequently removed over the SC and the pool filled with paraffin oil. The sciatic nerve was exposed for stimulation in-continuity in the popliteal fossa, and the sural nerve freed and sectioned distally for stimulation. Sciatic nerve stimulation involved low and medium threshold fibres from muscle and skin, whereas stimulation of the sural nerve provided solely a cutaneous input. We did not stimulate at C-fibre strengths for the comparison of WT and KO mice because preliminary experiments failed to reveal consistent reliable field potentials in recordings from the cord dorsum. In the rat, the antecedent volleys in the lower threshold A-fibres can inhibit C-fibre evoked field potentials which can be revealed better using TTX to block A-fibres leaving conduction intact for a group of TTX-resistant unmyelinated fibres [38]. However, the procedures were considered impractical to allow a valid comparison of WT and KO mice with the limited numbers of mice available for this part of the study.

Stimulation was via bipolar Platinum hook electrodes with 0.05 or 0.1 ms square wave voltage pulses (Digitimer DS2) at 2 Hz and the intensity set at 5×threshold (5T) for the systematic comparison of WT and KO mice. A plastic ensheathed 100 µm diameter Pt. wire was used for focal recording at the cord dorsum surface with the reference electrode on nearby muscle. The spatial distribution of the evoked potentials was then determined at the root entry zone (REZ) in 200 µm steps from the rostral end of T13 to the caudal end of L2 vertebral levels (the location of the epicentre of the hind limb representation; cf. [39]). At each site, 20 evoked responses were recorded through a FET-follower head stage and fed to a high-gain AC-couple differential amplifier (Digitimer DL150) with HF bandwith at 10 or 3 kHz and TC 30 ms. Signals were fed to a CED interface, sampled at 20 kHz and the average of 20 responses derived for each site using Spike2 software.

### Statistical Analysis

Data are expressed as mean ± S.E.M. Behavioural, biochemical and immunohistochemical studies were statistically analyzed with one-way or two-way analysis of variance (ANOVA; for repeated measures for behavioural tests) followed by post-hoc multiple comparisons testing with the Holm-Sidack method. ANOVA on ranks was used when the data failed the normality or equal variance tests. t-test or Mann-Whitney U-test as appropriate were employed to compare two groups. A p value <0.05 was considered to be statistically significant. All statistical analyses were performed using “SigmaStat for Windows” (version 2.0; Jandel Corporation, San Rafael, CA, USA). For the electrophysiological data, a mixed design two-way ANOVA (SPSS statistical software) was used with the between factor set as WT or EphB1KO and the within factor as level of the spinal cord. Additional post-hoc analysis (independent t test or Mann-Whitney Rank Sum test) was done at each position along the rostro caudal axis.

## Results

### EphB1 KO Mice Respond Normally to Acute Pain Stimuli and their Small Diameter Primary Sensory Afferents are Anatomically Intact, Although Changes in Connectivity of Larger Diameter Cutaneous Fibres were Observed

#### Locomotor coordination

A prerequisite for any behavioural study of an animal’s susceptibility to noxious and non-noxious stimulation in models of chronic pain is an intact functioning motor system. Therefore, prior to the systematic use of a number of established tests of acute nociception to assess the functional integrity of primary nociceptive afferents, we examined proprioception, coordination and tactile sensibility in WT and KO mice as an aspect of locomotion using the Rotarod test. (figure 1A). The scores revealed no clear differences between the two groups save perhaps for a slight trend towards a reduced performance in the KO mice at lower rotation speeds, but this did not reach significance.

*Acute thermal nociception* was assessed using a radiant heat source applied to the plantar surface of the hind paw (Hargreaves test) or a hot plate (figure 1B–C). *Mechanical sensory thresholds* were assessed manually using von Frey hairs (figure 1D). Neither acute thermal nociception or the response to mechanical stimulation differed significantly in WT and KO mice. These behavioural results confirmed our previous finding of a lack of involvement of EphB1 receptors in acute nociception, and indicated the functional integrity of nociceptive pathways, despite the absence of functional EphB1.

#### Immunohistochemical analysis of primary sensory afferents

We further confirmed the anatomical integrity of primary sensory afferents by immunohistochemical analysis of lumbar DRGs and nociceptive terminals in the dorsal horn of the spinal cord. DRGs were double-stained with a general neuronal marker (PGP9.5 or beta-tubulin) and a marker for either peptidergic or non-peptidergic nociceptive neurons, respectively, using anti-CGRP antibodies or the isolectin IB4. The proportions of DRG neurons stained with the different markers were calculated by counting both the total number of nucleated neurons, and the number of CGRP or IB4- positive neurons, present in randomly chosen sections. There were no differences between wild type and EphB1 KO mice in the proportion of DRG neurons stained with the two different markers (table 1). Similarly, nociceptive afferent terminations were examined after staining lumbar spinal cord sections with anti-CGRP antibodies and the isolectin IB4. Again there was no significant difference in the area occupied by nociceptive afferents in the dorsal horn of the spinal cord in wild type and EphB1 KO mice (table 1), suggesting anatomical integrity of these primary afferents terminations in the mutant mice, in agreement with their normal acute nociception (figure 1B–C).

**Table 1:** Lack of alteration in DRG neurons and dorsal horn immunoreactivity in EphB1 KO mice.

Table 1		WT (n = 3)	EphB1 KO (n = 3)
**DRGs**	% of CGRP positive neurons	31.8+/−0.01	30.6+/−0.04
	% of IB4 binding neurons	34.5+/−0.021	34.9+/−0.03
	% of N52 stained neurons	35.8+/−0.027	36.7+/−0.038
**Dorsal Horn**	Area covered by CGRP-immunoreactive terminals (µ^2^)	38899.8+/−1528.489	39645.3+/−884.572
	Area covered by IB4-binding terminals (µ^2^)	24026.1+/−1472.583	24325.3+/−440.958

Sections of lumbar L4 and L5 DRGs were double-stained with markers for the different subpopulation of neurons in the DRG and a general neuronal marker (PGP9.5 or beta-tubulin). Anti-CGRP antibodies and the isolectin IB4 bind respectively to peptidergic and non-peptidergic small and medium sized nociceptive neurons, whereas N52 antibodies bind to high molecular weight neurofilament present in large mechanoceptive and proprioceptive neurons. Quantification of the proportion of the total number of neurons (identified by the general neuronal marker), which also stained for a specific subpopulation marker, was carried out. No difference was observed between WT and EphB1KO mice. Sections of lumbar spinal cords were stained with anti-CGRP antibodies of the isolectin IB4, which bind respectively to the terminals of peptidergic and non-peptidergic nociceptive afferents. No difference was observed between WT and EphB1KO mice in the area covered by CGRP-positive and IB4-positive terminals in the dorsal horn of the spinal cord.

Large mechanoceptive and proprioceptive DRG neurons were stained with N52 antibodies, which recognise high molecular weight neurofilament and again there was no difference in the proportion of neurons in the DRGs between WT and EphB1 KO mice (table 1) in agreement with their normal mechanical sensitivity and normal motor behaviour, which requires intact vestibular, propriospinal and tactile sensation (Figure 1A and 1D).

#### Electrophysiological comparison of afferent transmission in WT and KO Eph.B1 mice

**Figure 2:**
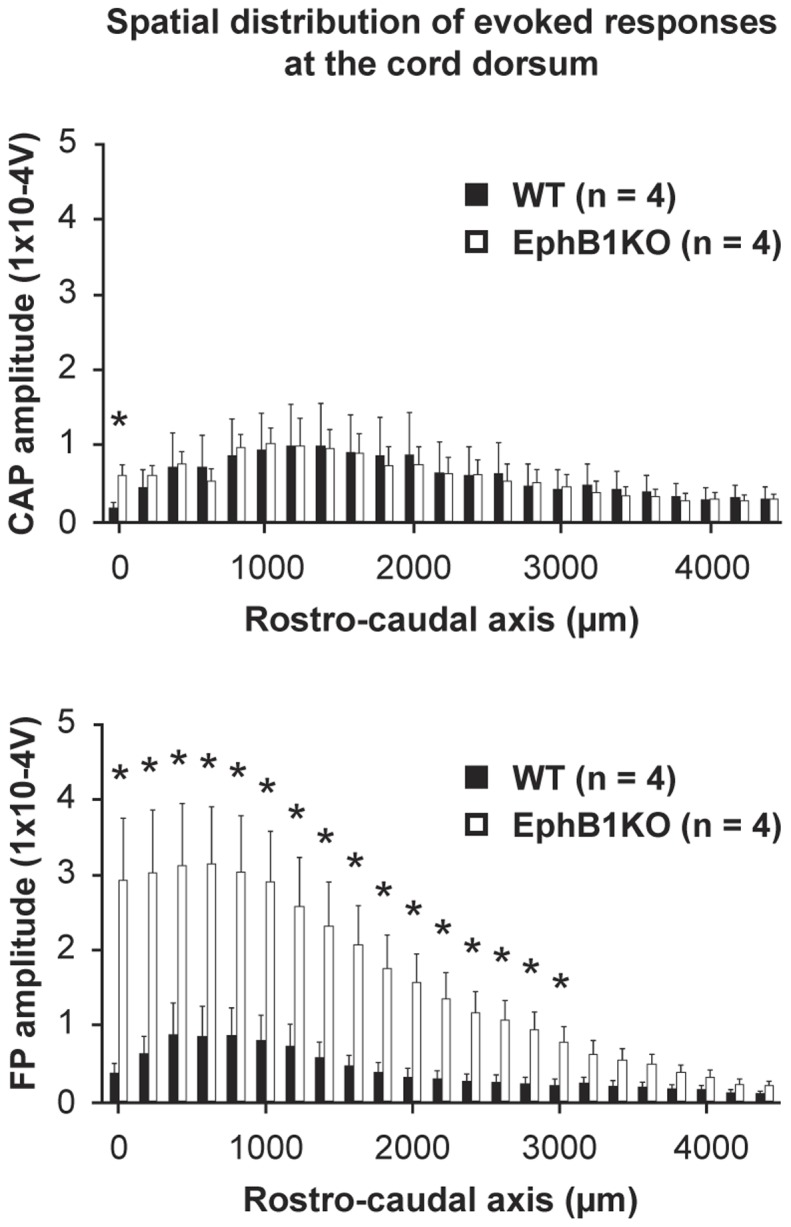
EphB1 KO mice display enhanced A-fibre evoked field potentials along the cord dorsum. Electrical stimulation of the central end of the cut sural nerve at 5T typically evoked a synchronous triphasic compound action potential (CAP), followed by a negative field potential (FP) at the root entry zone. Recordings were made systematically along the rostro-caudal axis 200 µm apart. At each site, 20 successive responses were averaged and the amplitudes (Voltsx10-4) plotted as a function of their rostro-caudal distribution. CAP values were normalised (amplitude and distribution) so allowing comparison of resulting FPs to inputs of similar strengths. Top and bottom panels respectively show the spatial distribution of the CAP and FP amplitudes, with the mean evoked-potentials at each successive position indicated by the paired histograms (WT: filled column; EphB1KO: empty column). While no statistical difference was found between genotypes in the amplitude of the evoked-CAPs (mixed design two-way ANOVA, between factors, p>0.05), the distribution of the evoked-FPs (bottom panel) along the rostro-caudal axis of the cord was significantly different between WT and EphB1KO mice (between factors, p<0.001), indicating that knocking out the EphB1 gene produced profound alterations in the amplitude of Aβ/Aδ fibre-mediated synaptic responses of the dorsal horn neurones in EphB1KO mice. For both types of evoked-potentials, a significant effect (p<0.001) was present for the within factor (level of the spinal cord) indicating that they differ along the rostro-caudal axis of the cord within each genotype. As indicated (stars), post-hoc analysis (independent t -test or non-parametric Mann-Whitney rank sum test) showed that, while it is only the case at the first rostral position for the CAPs, the majority of the FPs were significantly different between the two genotypes (p<0.05), except for the most caudal sites (3200 to 4000 µm) where the signals appeared greatly reduced both for WT and EphB1KO mice.

Further evidence concerning the functional status of primary afferent pathways in these KO mice relates to the somatotopic organisation and synaptic transmission to 2^nd^ or higher order neurones. This was determined electrophysiologically by measuring the spatial distribution and amplitudes of the compound action potentials (CAPs) and field potentials (FPs) evoked at the cord dorsum by electrical stimulation of the sciatic and sural nerves (figure 2). For comparison of the WT and KO mice the stimulus intensities were set at 5× T where T is the threshold of the lowest threshold fibre in the stimulated nerve conducted to the root entry zone. For the sciatic nerve this threshold is that of the fast conducting primary afferent fibres (Group1A) from muscle spindles in the calf and foot, whereas for the sural nerve the threshold is that of Aβ fibres cutaneous fibres innervating the lateral plantar surface of the foot. The epicentre of the hindlimb representation lay under the L1 vertebra. No significant differences between WT and EphB1KO mice were observed either in the spatial profile or peak amplitudes of the evoked-CAPs and FPs with stimulation of the sciatic nerve (p>0.05; data not shown), nor in the evoked-CAPs with stimulation of the sural nerve (figure 2A). Interestingly, however, with stimulation of the sural nerve the peak amplitudes of the FPs were highly significantly increased by approximately threefold in the KO as compared to WT (P<0.001; figure 2B) and this was manifest over the entire spatial array of recordings. This suggests that the lack of EphB1R during development resulted in changes in spinal connectivity of the large and medium-sized cutaneous Aβ/δ fibres in the superficial dorsal horn.

### Persistent Pain Behaviour Following Tissue Injury is Significantly Reduced in EphB1 KO Mice

**Figure 3:**
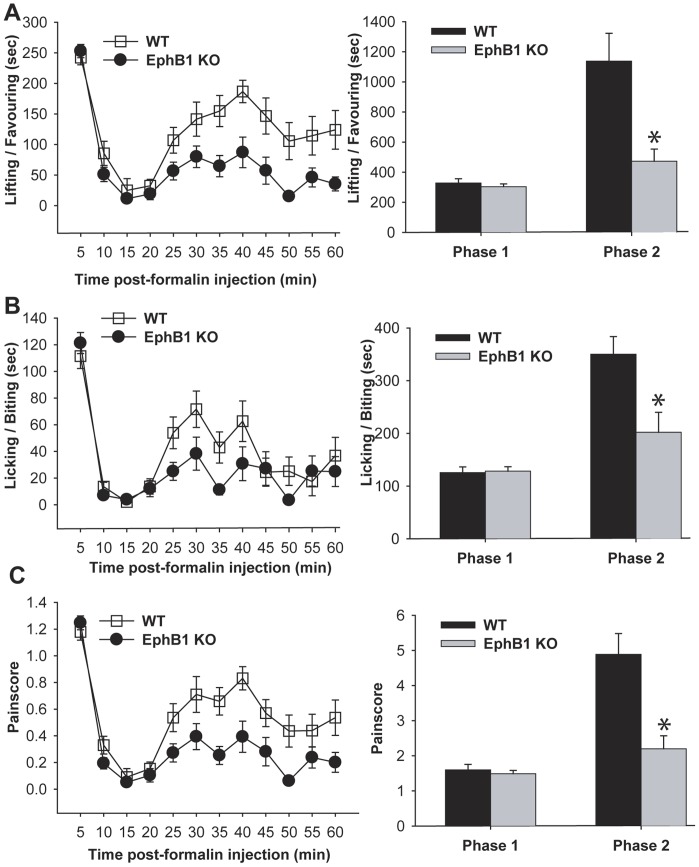
EphB1 KO mice display less spontaneous pain behaviour than WT mice in the formalin test. The behavioural responses of EphB1 KO and WT mice were examined in the formalin test. A formalin solution was injected in one of the hind paws, and the mice were observed for 60 minutes. Pain-related behaviours (lifting-favouring and licking of the injected paw) were recorded in 5 minutes intervals, and the results are graphically represented in panels A and B. A ‘pain score’ was calculated based on these data, and is represented in panel C. The animals displayed initially an acute response to the injection (Phase I), which is akin to acute nociception and depends upon activation of peripheral terminals of nociceptors. This phase lasted approximately 10 minutes. After a pause of a few minutes, during which little pain related behaviour was observed, the mice began again to lift or lick the affected paw (Phase II). When the cumulative results of EphB1 KO and WT mice for Phase I and Phase II were compared, there was no difference between WT and KO during Phase I. However, EphB1 KO mice displayed significantly less pain related behaviours (Lifting/favouring, licking and pain score) as compared to WT mice during Phase II. Bar charts represent mean +/− SEM Asterisks indicate a significant difference WT vs EphB1 KO with p<0.05 (t-test). WT n = 13, EphB1 KO n = 15.

We then tested WT and EphB1 KO mice in a model of persistent pain, the formalin test. Spontaneous pain behaviour elicited by the injection of a diluted solution of formalin in the plantar surface (i. pl.) of the hind paw was recorded for 60 min. We recorded both licking and lifting/favouring of the injected paw, which we then combined to obtain a pain score (figure 3). The response following formalin injection can be divided in two phases. Phase I, corresponding to acute nociception, lasts about 10 minutes, and during this phase no significant difference was observed between WT and EphB1 KO mice. A short interval then occurs, during which the animal displays no signs of spontaneous pain, followed by Phase II, the resumption of pain behaviour, lasting for 30 or more minutes, depending on mouse strain. In part, Phase II is attributed to central sensitisation. EphB1 KO mice displayed significantly less spontaneous pain behaviour in Phase II of the formalin test as compared to their WT counterparts (p<0.05).

### EphB1 Receptor Expression is Required for the Onset of Short-term Inflammatory Pain and EphB1 Deficient Mice Recover Significantly Faster from Thermal Hyperalgesia and Mechanical Allodynia in a Long-term Model of Inflammation

Peripheral tissue inflammation can lead to the onset of persistent (chronic) pain, mediated by mechanisms including peripheral and central sensitisation.

**Figure 4:**
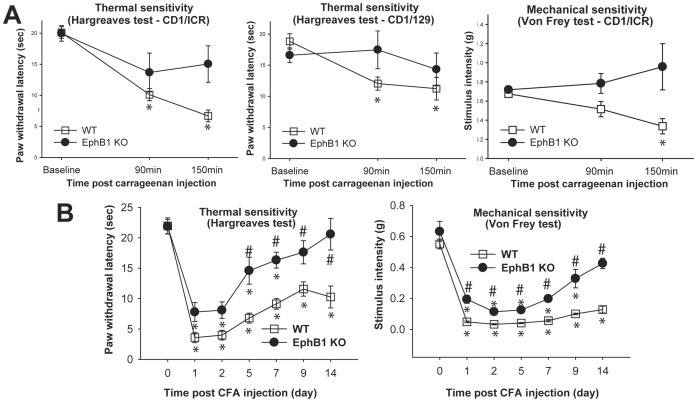
EphB1 KO mice display reduced thermal and mechanical hyperalgesia in inflammatory pain models. The behavioural responses of EphB1 KO and WT mice were examined in two test of inflammatory pain. Either carrageenan (A) or CFA (B) were injected in a hind paw, and responses to thermal (Hargreaves) and mechanical (von Frey) stimuli were recorded up to a maximum of 150 minutes (A) or 14 days (B). Thermal sensitivity following carrageenan injection was tested in mice of two genetic backgrounds, CD1/ICR and CD1/129, and mechanical allodynia was tested in CD1/ICR mice only. All WT mice developed thermal and mechanical hyperalgesia (p<0.05). EphB1 KO mice did not develop thermal or mechanical hyperalgesia. Following CFA injection, which elicits a greater and more prolonged inflammatory response as compared to carrageenan, both WT and EphB1 KO mice developed thermal and mechanical hyperalgesia (B). However, EphB1 KO mice displayed a faster recovery in terms of thermal hyperalgesia and a significantly smaller development of mechanical allodynia. * p<0.05 vs respective baseline, # p<0.05 comparing WT and EphB1 KO mice at equivalent time points (Repeated Measures ANOVA followed by Multiple Comparisons versus Control Group, Holm-Sidak method). A) CD1/ICR Hargreaves WT n = 9, EphB1 KO n = 9, von Frey WT n = 10, EphB1 KO n = 10; CD1/129 WT n = 8, EphB1 KO n = 6; B) n = 8 for all groups except EphB1 KO in the von Frey test, where n = 9.

We used two tests of inflammatory pain, of shorter and longer duration (carrageenan and CFA injection in the hind paw respectively), to assess the role played by EphB1 receptor in the onset of inflammatory pain. Thermal and mechanical responses were measured before and after i.pl. injection with carrageenan and CFA (figure 4). The onset of both thermal hyperalgesia and mechanical allodynia was delayed and depressed in EphB1 KO mice in the carrageenan test (figure 4A). Thermal hyperalgesia was assessed in two strains of mice (CD1 and CD1/129), since it is well established that thermal and mechanical sensitivities depend on genetic background in mice. The results were similar, even if prevention of thermal hyperalgesia was more complete in the strain which developed a less pronounced hyperalgesia in the WT mice (CD1/129, figure 4A). The latency to paw withdrawal to thermal stimuli was significantly decreased already at 90 min after carrageenan injection in WT animals for both strains (p<0.05), whereas there was no significant decrease in EphB1 KO, again regardless of strain. In the CD1 strain of mice tested only the WT mice developed allodynia (p<0.05), none being present in the EphB1 KO.

In the longer duration CFA injection model, responses to thermal and mechanical stimuli were recorded at 1, 2, 5, 7, 9 and 14 days after CFA injection (figure 4B). Both WT and EphB1 KO mice developed thermal hyperalgesia, but whilst the latency to paw withdrawal remained significantly lower than baseline values for the duration of the experiment in WT (p<0.05), by day 9 in EphB1 KO mice these latencies had returned to values not significantly different from baseline. Both WT and EphB1 KO developed significant (p<0.05) and persistent mechanical allodynia, but the thresholds remained significantly lower for WT animals at 9 and 14 days (p<0.05), again indicating a faster recovery for EphB1 KO from day 9.

### The Increase in the Number of c-fos Expressing Cells in Laminae I and II of the Dorsal Horn, which Follows i.pl. Injection with Formalin or Carrageenan, is Significantly Smaller in EphB1 KO Mice

**Figure 5:**
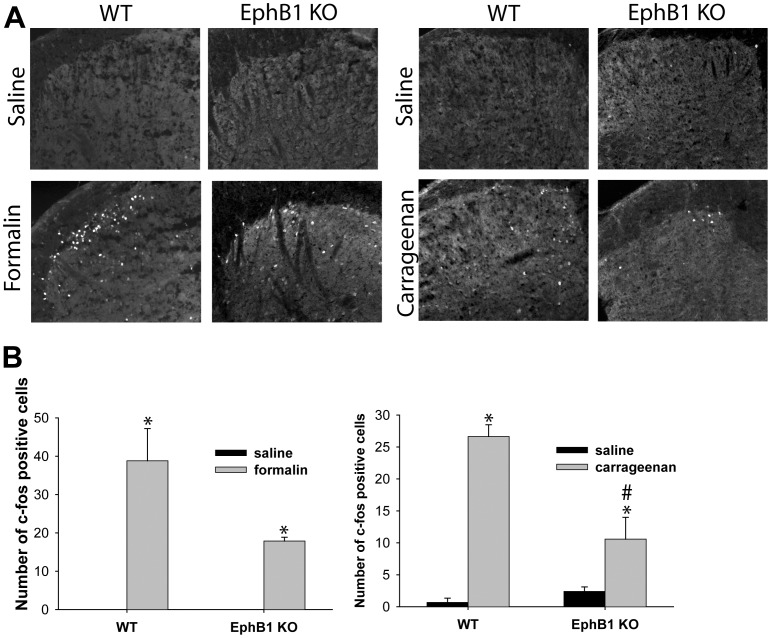
Upregulation of c-fos expression following formalin or carrageenan injection is reduced in EphB1 KO mice. C-fos immunoreactivity was examined in EphB1 KO and WT mice following formalin and carrageenan injection. Saline injected animals were used as controls. (A) photomicrographs of section of lumbar spinal cord of EphB1 KO and WT mice injected with formalin, carrageenan or saline as indicated, immunostained with anti c-fos antibodies. B) c-fos positive cells were counted in lumbar cord section of mice treated as in (A). Saline treated animals had none or few c-fos positive cells. Both formalin and carrageenan injection induced a significant increase in the number of c-fos positive cells in WT mice (two-way ANOVA followed by multiple comparisons, Holm-Sidak method, for carrageenan, t-test for formalin experiments), but carrageenan or formalin injection caused a significantly lower increase in EphB1 KO mice. * p<0.05 vs respective saline, # p<0.05 vs WT. For all experimental groups n = 3.

C-fos expression in dorsal horn neurons is considered as an indicator of activation of nociceptive pathways. We counted the number of c-fos expressing neurons in laminae I and II of the dorsal horn in immunostained sections of spinal cord of animals, which had received a unilateral i.pl. injection of saline, formalin or carrageenan (figure 5). A large and significant (p<0.05) increase in the number of c-fos expressing neurons was observed in WT mice following formalin and carrageenan injection, as compared to saline treated WT mice. A significant increase in c-fos expression was also present in formalin or carrageenan treated EphB1 KO mice, but this was significantly smaller than in WT (p<0.05).

### The NR2B Subunit of the NMDA Receptor is Phosphorylated in WT, but not EphB1 KO Mice following Formalin or Carrageenan Injection

**Figure 6:**
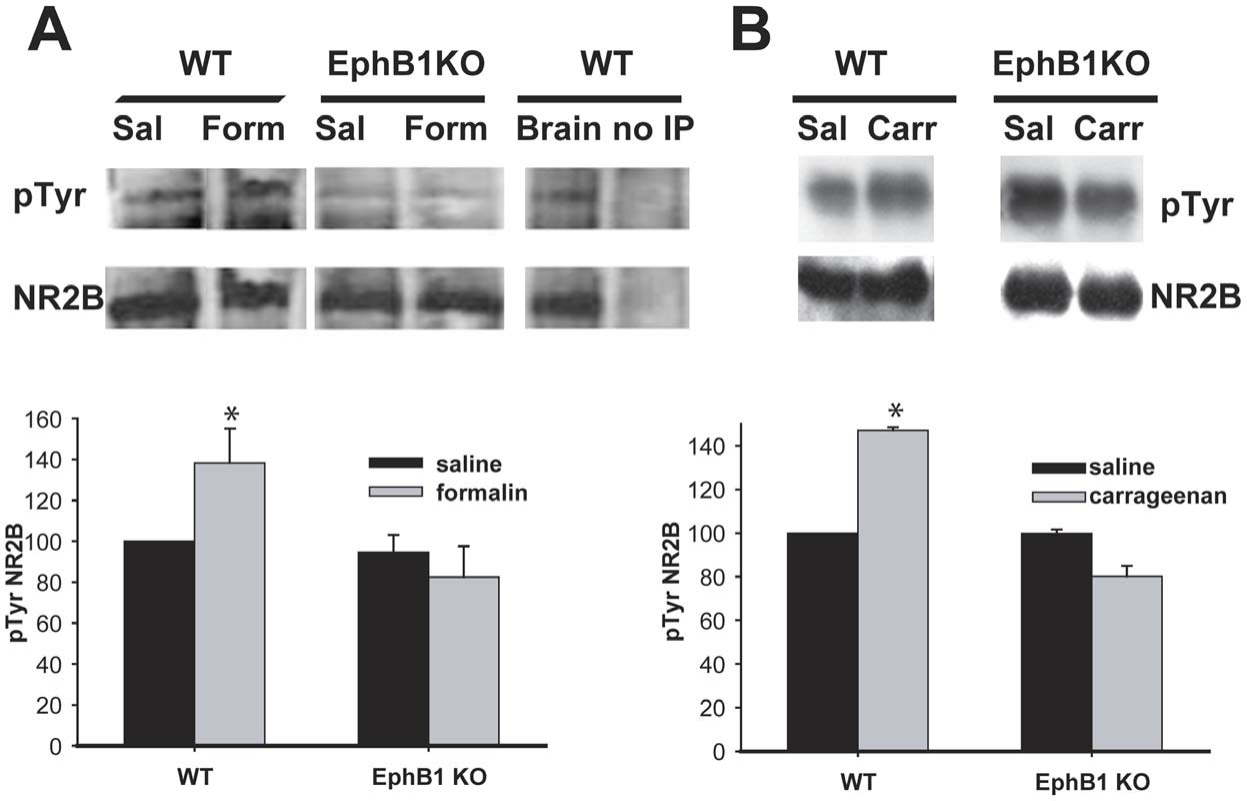
Lack of increase in NR2B phosphorylation in EphB1 KO mice following formalin or carrageenan injection. Tyrosine phosphorylation of the NR2B subunit of the NMDA receptor was analysed by immunoprecipitation followed by Western Blotting in WT and EphB1 KO mice after injection with formalin or carrageenan. Saline injected animals were used as controls. Spinal cords were dissected from injected animals 1 h (A) or 3 hours (B) after injection. Representative immunoblots are shown in the top part of panels A and B. In panel A, the last two columns represent positive (mouse brain tissue) and negative (immunoprecipitation of mouse brain tissue conducted in the absence of anti-NR2B antibodies) controls of the immunoprecipitation and Western blotting protocol. The blots were quantified, and the ratio of phosphoTyr to the total NR2B present was calculated. The bar charts in the bottom half of panels A and B show the mean and SEM of the levels of phosphoNR2B present in EphB1 KO and WT mice following injection with Formalin, Carrageenan or saline, as indicated. There was a significant increase in Tyrosine phosphorylation of NR2B in WT mice following formalin (A) and carrageenan injection (B), p<0.05. There was no change in the level of NR2B phosphorylation in EphB1 KO mice following either injection. Comparisons performed with t-test for WT in the formalin experiment and EphB1 KO in the carrageenan experiments, with Mann-Whitney U-test in the remaining experiments. * p<0.05 vs respective saline. For all experimental groups n = 3, except in A) EphB1 KO saline n = 4.

To examine further the role of the NMDA receptor in EphB/ephrinB signalling, we measured the phosphorylation state of its NR2B subunit in spinal dorsal horn preparations of WT and EphB1 KO mice, after i.pl. injection with saline, formalin carrageenan (figure 6). NR2B was immunoprecipitated from the spinal cords, and western blotting was performed using anti-phosphotyrosine antibodies, followed by anti-NR2B antibodies to measure total NR2B. Levels of phosphorylation were calculated using densitometry. Both carrageenan and formalin injection caused a significant increase in tyrosine phosphorylation of the NR2B subunit in WT (p<0.05), but not in EphB1 KO mice.

### Deletion of the EphB1 Receptor Gene Reduces, but does not Abolish, Mechanical Allodynia in Two Models of Neuropathic Pain and Thermal Hyperalgesia in the Seltzer Model

**Figure 7:**
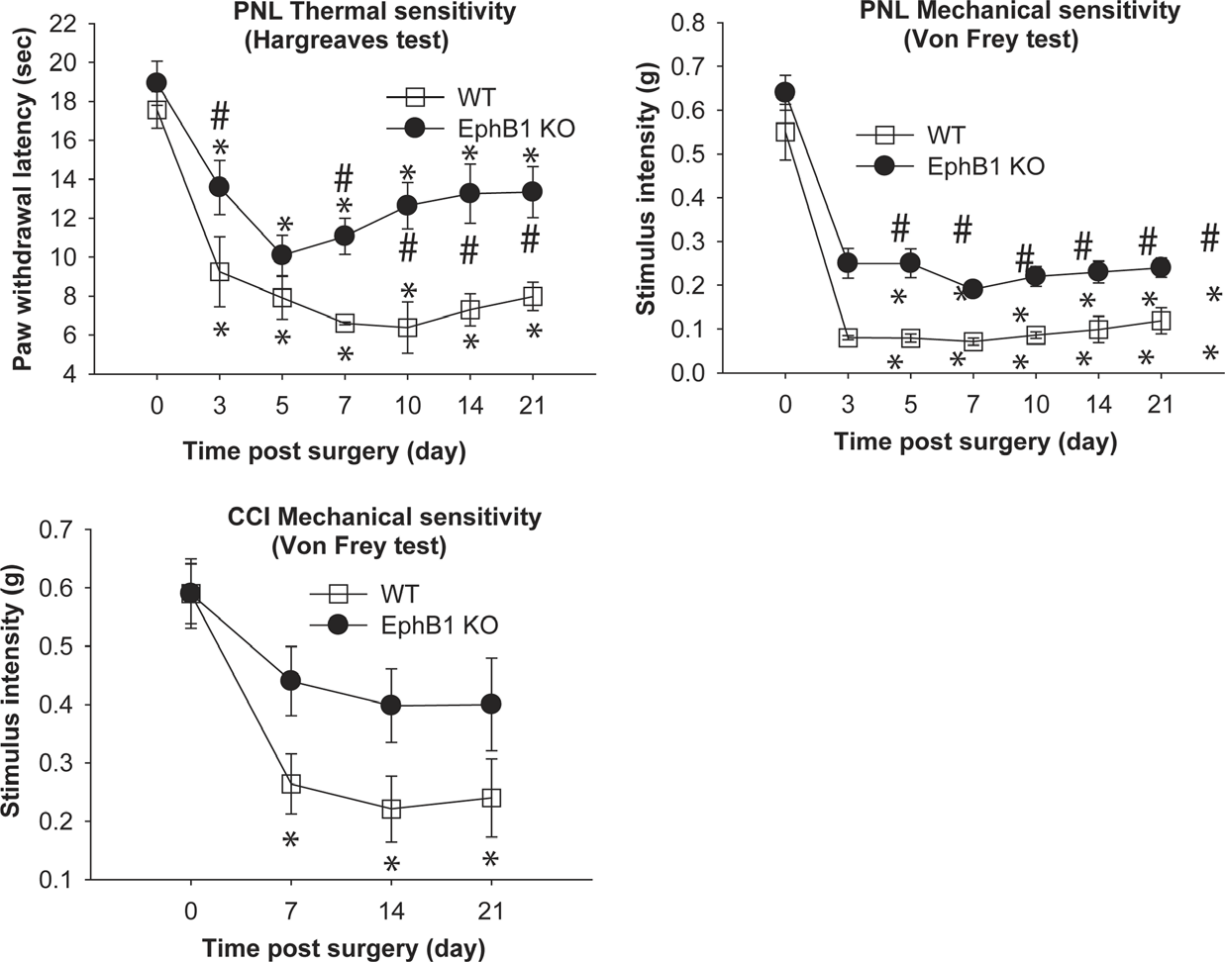
Accelerated recovery from mechanical and thermal hyperalgesia following nerve injury in EphB1 KO mice. Behavioural responses following peripheral nerve lesion were examined in EphB1 KO mice and WT mice. Two models of nerve injury were used, partial nerve lesion (PNL) and chronic constriction injury (CCI). Development of both thermal and mechanical hyperalgesia were studied after PNL, mechanical hyperalgesia only was examined after CCI. WT mice developed significant thermal hyperalgesia in both models. In WT mice with PNL, hyperalgesia developed by 3 days after injury and persisted throughout the duration of the experiment. This reduction in paw withdrawal latency was significantly smaller in EphB1 KO mice at all time points, except 5 days after injury. WT mice also developed mechanical allodynia with a marked decrease in stimulus intensity required to trigger a lifting response of the injured paw as compared to baseline. EphB1 KO mice displayed a less pronounced mechanical allodynia when compared to WT at all time points (p<0.05). In the CCI model mice responses to mechanical stimuli were tested at 7, 14 and 21 days after injury. WT mice developed a significant mechanical allodynia by 7 days (p<0.05), and this persisted throughout the experiment, whilst EphB1 KO mice failed to develop a significant mechanical allodynia.* p<0.05 vs respective baseline, # p<0.05 vs WT. Test: repeated measures ANOVA followed by multiple comparisons, Holm-Sidak method. PNL WT n = 6, EphB1 KO n = 9; CCI WT n = 7, EphB1 KO n = 5.

We examined the development of mechanical allodynia in two models of neuropathic pain, the PNL and CCI models (figure 7). The CCI model has a more marked inflammatory component.

Animals with PNL were tested 3, 5, 7, 10 14 and 21 days after injury. Both genotypes developed thermal hyperalgesia following injury, but its development was significantly less marked in EphB1 KO mice. In WT mice, thermal hyperalgesia developed from the first day of testing (3 days after injury) and persisted throughout the duration of the experiment. In EphB1 KO mice, the reduction in paw withdrawal latency was significantly smaller than in WT mice at all time points, except 5 days after injury. The mice also developed signs of mechanical allodynia with a marked decrease in the threshold of the response (lifting response to tactile stimulation) of the injured paw as compared to baseline (see figure 7). This increased mechanical sensitivity was markedly reduced in EphB1 KO mice when compared to WT at all time points (p<0.05).

A more complete prevention of development of mechanical allodynia was observed in the CCI model. Animals were tested at 7, 14 and 21 days after injury. WT mice developed a significant mechanical allodynia by 7 days (p<0.05), and this persisted throughout the experiment, whilst EphB1 KO mice failed to develop a significant mechanical allodynia. Note that autotomy was not observed in any of the animals. Sham operated animals did not develop significant hyperalgesia or allodynia in either genotype (results not shown).

For CCI injured mice, we verified that the behavioural difference between WT and KO mice was not due to differences in the extent to which the animals were affected by the injury: we stained DRG neurons with ATF3 (a marker of axonal injury), and IB4 (a marker for small, non peptidergic, nociceptors), and sections of dorsal horn were stained with IB4. In both genotypes, CCI caused a similar and significant (p<0.05) increase in the number of DRG neurons with ATF3+ nuclei in the injured compared to the uninjured side (uninjured, 0.09+/−.057 for WT and 0.109+/−.0711 for EphB1 KO; injured, 15.5+/−1.231 for WT and 12.1+/−4.113 for EphB1 KO). There was no change in the proportion of IB4+ neurons in the injured DRG, but a loss of IB4+ terminals in lamina II of the dorsal horn was observed, in a lateral position (data not shown). C-fos activation in the dorsal horn was absent at 28 days after injury in both genotypes (data not shown).

### Microglial Activation in the Spinal Cord following PNL is Attenuated in EphB1 KO Mice

**Figure 8:**
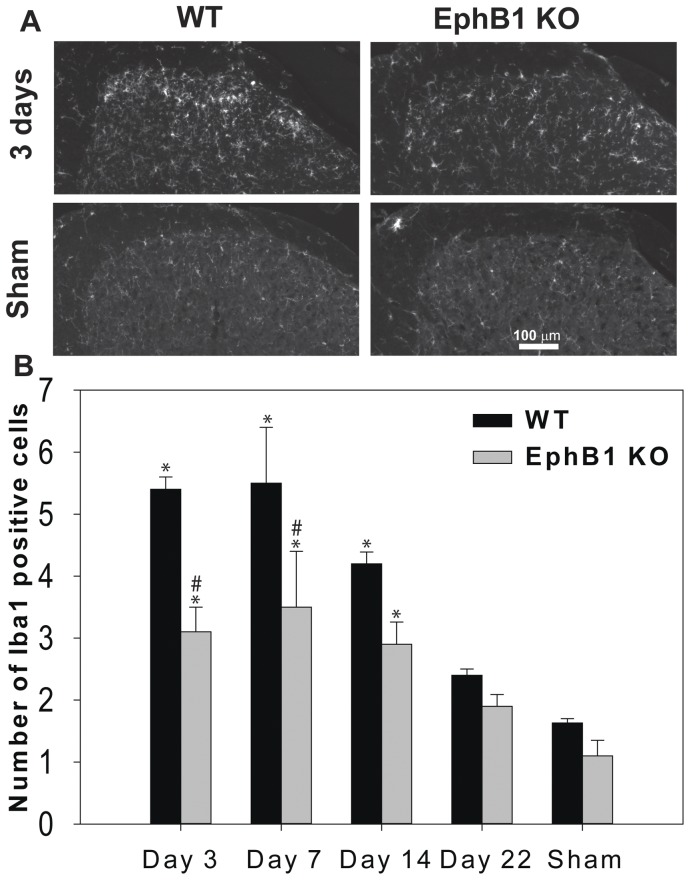
Microglial activation following PNL is attenuated in the spinal cord of EphB1 KO mice. In (A) representative immunostaining for Iba1 in the superficial laminae of the dorsal horn of the spinal cord 3 days after PNL, or in sham operated animals. The hemicord ipsilateral to the sciatic nerve partial ligation is shown. In (B) bar chart illustrating cell counting for Iba1 positive cells in the dorsal horn. An increase in microglia cell activation was shown after PNL in both WT and EphB1 KO mice at 3, 7 and 14 days after lesion (p<0.05). However, the increase of immunoreactivity for Iba1 was attenuated in EphB1 KO mice (p<0.05 overall, and at 3 and 7 days after lesion). Data shown as mean ± SEM. * p<0.05 vs respective sham, # p<0.05 vs WT. Test: two-way ANOVA followed by multiple comparisons, Holm-Sidak method. WT and EphB1 KO n = 3 for each timepoint.

Microglial cell activation has been shown to be associated with the onset of neuropathic pain. We identified microglial cells with Iba1 antibodies and manually counted the number of morphologically identified activated microglial cells in the dorsal horn of the spinal cord in control, sham animals, and in animals which had undergone PNL, both ipsilaterally and contralaterally to the lesion. In sham animals there was no difference in the number of activated microglial cells in WT or EphB1 KO mice (figure 8 A, B). Following nerve injury, increased microglia activation was observed in the ipsilateral dorsal horn of the spinal cord from day 3 onwards. This activation was present also in EphB1 KO mice, but to a significantly lesser extent, as compared to WT (p<0.05). By day 22, though, there was no significant microglial activation, as compared to sham animals, in either WT or KO mice. We did not observe a significant microglial activation on the contralateral side (average number of activated microglial cells/100 µm^2^: day 3 WT 1.133+/−0.0726, EphB1 KO 1.117+/−0.0167; day 7 WT 1.083+/−0.209, EphB1 KO 1.400+/−0.277; day 14 WT 1.258+/−0.0902, EphB1 KO 1.175+/−0.142; day 22 WT 1.046+/−0.123, EphB1 KO 0.917+/−0.0928).

## Discussion

In this study we characterised extensively EphB1 receptor KO mice in terms of their responses to acute noxious stimuli and susceptibility to persistent (chronic) pain, in a number of models in which spontaneous pain behaviour and/or thermal and mechanical hyperalgesia have been attributed at least in part to the development of central sensitisation, an activity-dependent alteration of synaptic connectivity within the spinal cord,. Our main findings are 1) the EphB1 receptor is not essential for the development of peripheral nociception, nor for thermal or mechanical sensitivity; 2) functional EphB1 receptors are required for the development and/or maintenance of thermal and mechanical hyperalgesia and spontaneous pain in a variety of pain models; 3) EphB1 receptors are necessary for the induction of phosphorylation of the NR2B subunit of the NMDA receptor and are involved in the increase of c-fos expression in models of inflammatory pain and tissue injury and in microglial activation following PNL.

EphB1 receptors, together with other Eph receptors, play an important role in neuronal development (see e.g. [40]), therefore it was important to establish that EphB1 KO mice were normal in terms of development of nociceptive pathways and nociception, in order to be suitable in models for the study of the modulation of pain processing. This had not been examined in previous studies using these mice. Since pain sensitivity in animal models is measured through behavioural tests dependent on a motor response, it was also important to test naïve KO mice in comparison to WT littermate in a motor test, particular in light of findings indicating that EphB1 KO exhibit neuronal loss in the substantia nigra [41]. We found no indication that this loss affected the response to acute noxious stimuli using a variety of tests, including thermal and mechanical stimuli, or to the first phase of the formalin test, reflecting acute tissue damage. Gross anatomical abnormalities were also absent from both the DRG and the dorsal horn of the spinal cord. Similarly electrophysiological recording at the cord dorsum, measuring the spatial distribution and amplitudes of the CAPs and FPs following sciatic nerve stimulation, did not reveal any difference between WT and EphB1 KO mice in terms of somatotopic organisation of sensory projections in the spinal cord. Although the presence of developmental defects in glutamatergic synapses in the hippocampus of transgenic EphB2 KO mice remains unclear [42], [43], it is possible that in single mutants (EphB1 or EphB2 receptors KO) compensatory mechanisms, due to the presence of other EphB receptors, which bind promiscuously to the same ephrinB ligands, may allow a comparatively normal development. We cannot however exclude the presence of subtle changes, for example at the level of the glutamatergic synapses of sensory afferents onto dorsal horn neurons, below the level of detection behaviourally. In this respect our finding of enhanced sural nerve evoked superficial dorsal horn FPs is of relevance in that it indicates such a change in the connectivity of Aβ-fibres. Our simplest explanation for this finding draws on the changes observed early postnatally in the rat where the predominant A-fibre innervation of the superficial dorsal horn at birth undergoes a dramatic, activity-dependent withdrawal of the nerve terminals to deeper layers over the first postnatal weeks [44], [45]. It is proposed here that such functional and structural reorganisation did not occur in EphB1KO mice. Further studies are needed to determine whether inhibitory or excitatory interneurons, projection neurons or both, are involved in this refinement process. Although this change in the connectivity of A fibres in the sural nerve is of considerable interest in view of the guidance role of Eph receptors during development, it had no demonstrable impact on the behavioural tests we employed, or on the gross anatomical connectivity of C and Aδ fibres. Another possible explanation for the enhanced synaptic transmission we observed could be a change in the electrophysiological properties of the large diameter Aβ-fibre, as described in DRG neurons for a rat model of osteoarthritic pain [46] and more recently in a neuropathic pain model [47].

Clearly indicated for future work would be a comparison of a), the cord dorsum CAP and field potentials evoked by fibres in the sural and saphenous nerves; b), their depth profiles in the dorsal horn as this would provide direct evidence of a developmentally – based shift in their sites of termination and/or or their synaptic strength.

Following our initial work demonstrating an involvement of EphB receptors-ephrins interaction in modulation of pain processing in the spinal cord [7], a growing number of other studies has provided evidence supporting a role of EphB receptors in a variety of short-term inflammatory and longer-term, chronic pain models (carrageenan and formalin injection in the hind paw, chronic constriction injury, pain induced by morphine withdrawal in dependent animals, cancer pain, see e.g. [9], [24], [48]–[52] ). Initially, the unequivocal identification of the specific EphB receptor involved had been impossible, due to the lack of suitable reagents and in particular specific agonists and antagonists. A study by Han et al. [25], performed on one of the strains of EphB1 KO mice used here, confirmed the hypothesis of an involvement of EphB1 receptors in neuropathic pain (thermal hyperalgesia in the CCI model) and in physical dependence to morphine, comparing the behavioural responses of EphB1 KO, heterozygous and WT mice. In their study, a complete lack of development of thermal hyperalgesia was observed in EphB1 KO mice in the CCI model. Here, we examine the EphB1 KO mice studied by Han et al. in a number of other models, including short-term (carrageenan injection) and long-term (CFA injection) inflammation, tissue damage and PNL. Even if the molecular and cellular mechanisms underlying hypersensitivity in experimental models of pain have not been completely clarified, it is clear that different molecular and cellular mechanisms underlie hypersensitivity in different pain models (see e.g. [23]), and also mechanical and thermal hyperalgesia in the same model; for example, with regard to neuropathic pain, it has been shown that there are significant differences in the mechanisms leading to chronic pain in different models [53], [54], and in the mechanisms leading to the induction of thermal and mechanical hyperalgesia. It is therefore important to establish if and to what extent EphB1 receptors contribute to thermal and mechanical hyperalgesia, as well as spontaneous pain behaviour, in pain states of different aetiology.

In a number of models (CCI and carrageenan injection) we observed a complete lack of development of mechanical and thermal hyperalgesia, as we expected on the basis of previous findings obtained in rats treated with the EphB receptor blocker EphB1-Fc (for carrageenan injection and thermal hyperalgesia in CCI; [7], [9]) or in EphB1 KO mice (for thermal hyperalgesia in CCI; [25]).

However, in the CFA-induced inflammatory pain model hyperalgesia and allodynia developed almost normally in KO mice, but recovery was accelerated. This is particularly interesting because due to its duration the CFA model would arguably be one of the most clinically relevant. Furthermore, this finding was partially replicated in the PLN model of neuropathic pain where thermal hyperalgesia developed almost normally in KO mice, but recovery was accelerated. The results obtained in the CFA and PNL models indicate that EphB1 receptors play a significant role in maintaining sensitisation hence emphasising their importance as a potential therapeutic target.

The behavioural findings reported here are evidence of a crucial role of EphB1 receptors in the onset and/or maintenance of thermal and mechanical hypersensitivity in a variety of models of pain, but what are the specific cellular and molecular mechanisms involved? Notwithstanding the number of differences in the molecular and cellular changes they induce, in all of the models we have used, a supposed common mechanism is represented by classical central sensitisation, an activity-dependent increase in the strength of synaptic transmission between primary sensory afferents and dorsal horn neurons [12]. Although such sensitisation is thought to be an NMDA receptor-mediated process, its mode of modulation is not yet fully understood. LTP at C-fibre synapses on a subpopulation of nociceptive neurons in lamina I of the dorsal horn could also contribute to pain amplification [14], [15]. Phosphorylation of NMDA receptor subunits has been proposed to lead to increased Ca++ entry through the receptor, both in central sensitisation and in NMDA dependent LTP [19], [55]. Liu et al. [56] showed that uncoupling Src from the NMDA receptor complex, thus preventing NMDA tyrosine phosphorylation, suppresses inflammatory and neuropathic pain. Phosphorylation of the NR2B subunit has been claimed to be important for the onset of persistent pain, however there is evidence both in favour and against NR2B involvement, particularly in inflammatory pain (in favour [8], [19], [20], [57], [57,58] [59]; against [18], [20], [60]).

Guo et al. [19] were the first to propose that NMDA receptor phosphorylation in central sensitisation could be modulated by other receptors, rather than simply by activation of the NMDA receptor itself (with consequent activation of Src tyrosine kinases); and they were also the first to show NR2B tyrosine phosphorylation in CFA induced inflammatory pain. Work from our laboratory had suggested a role for EphB receptors in central sensitisation, showing that, in analogy with what had previously been demonstrated in cultures of embryonic cortical and hippocampal neurons [27], EphB receptor activation could lead to NR2B subunit phosphorylation in the spinal cord, a process mediated by the activation of the non-receptor tyrosine kinase Src [8]. Furthermore, in a model of acute inflammatory pain (carrageenan injection) in adult rats [8] and also in the formalin model in adult mice [49], we showed NR2B tyrosine phosphorylation in the spinal cord following injection, in contrast to the findings of Caudle et al. [18], who found that the NR2B receptor is not involved in inflammatory pain. Liu et al. [52] replicated our finding showing that EphB receptor activation in rats induces NR2B phosphorylation, but also showed that blocking receptor activation with EphB2-Fc chimera (which blocks the activation of all EphB receptors) prevented the increased phosphorylation of NR2B in a model of cancer pain. On the basis of the findings reported in the literature, and discussed above, and in particular [7], [8], [27], [52], we hypothesised that in WT type mice inflammation or tissue injury may lead to activation of EphB1 in dorsal horn neurons, with consequent phosphorylation of NR2B; lack of functional EphB1 receptor would therefore abolish such increased phosphorylation. The findings reported here are consistent with the hypothesis that NR2B subunit phosphorylation is indeed downstream of EphB1 receptor activation in both the carrageenan model and in the formalin model, in which NR2B is tyrosine phosphorylated in WT but not in KO mice. However, our findings also provide support for the conflicting hypothesis [18],that NR2B phosphorylation is not required for the onset of chronic pain, since in some long-term models we observed the onset of hyperalgesia and allodynia also in EphB1 KO mice. We attempted to analyse NR2B phosphorylation in a longer duration model (CFA), using naive mice, and mice at 1, 7 and 14 days after injection of CFA or saline. Despite using 8 mice/genotype/timepoint (2 mice/sample), our results were inconclusive, with no obvious increase in NR2B phosphorylation in EphB1 KO mice, and a non-significant, 20% increase in phosphorylation in WT mice at 1 day only (data not shown). This would be consistent with the time course of NR2B phosphorylation in the spinal cord of rats, following CFA injection, reported by Guo et al. [19] but needs to be confirmed. The biochemical analysis of NR2B receptor phosphorylation in the mouse spinal cord is challenging, possibly due to low levels of the receptor as a proportion of total protein level; further investigations on the link between EphB1 receptors, NR2B phosphorylation and chronic inflammatory and neuropathic pain could probably more profitably investigated with genetic manipulation, for example by crossing EphB1 mutants with mice in which NR2B cannot be phosphorylated on tyr1472, which have been shown to develop attenuated neuropathic pain [20], or by developing a spatially and time restricted NR2B knockout, similar to the one generated for NR1 [17], [61]. This second alternative may give different results from those obtained in mutants in which tyr 1472 cannot be phosphorylated, since there are two other tyrosine phosphorylation sites on NR2B (tyr1252 and tyr 1336), which may play a role. It is however worth noting that NMDA-dependent synaptic plasticity in the hippocampus was shown to be independent of EphB2 receptor kinase activity, despite requiring the extracellular portion of the receptor [42], [43], in contrast to the requirement for kinase activity shown by Takasu et al. [27] in embryonic neurons in culture. It is therefore possible that EphB1 receptors may mediate alterations in spinal cord processing of pain independent of their kinase activity.

The immediate early gene c-fos has been shown to be expressed in neurons of the dorsal horn following activation of nociceptive pathways [62], [63], and Takasu et al. [27] showed increased c-fos expression following EphB receptor activation in cultured embryonic cortical and hippocampal neurons. We previously showed increase in c-fos-positive cells in the superficial laminae of the dorsal horn in carrageenan treated rats, an increase which was not present when EphB1-Fc was injected intrathecally prior to carrageenan injection, thus linking EphB1 receptor activation to c-fos expression also in vivo. Liu et al. [26] demonstrated increased c-fos expression in the dorsal horn of mice following high frequency stimulation of C fibres in vivo. This was prevented by EphB2-Fc pre-treatment and in EphB1 KO mice. Furthermore, Liu et al. [10] showed NR2B phosphorylation and increased c-fos expression in the dorsal horn in a morphine withdrawal model. Both increases were prevented by blocking EphB receptor activation with EphB2-Fc. Here, we show that both formalin and carrageenan injection induce an increase in c-fos expression in WT but a significantly smaller increase in EphB1 KO mice. This suggests that c-fos upregulation may mediate some of the effect of EphB1 activation on pain sensitivity. We cannot directly link the increase in c-fos expressing neurons to NR2B phosphorylation, since we cannot demonstrate that the neurons expressing c-fos are the same in which NR2B is phosphorylated. Hopefully technical improvement in immunohistochemical approaches will allow doing so in the future. However the findings by Takasu et al [27] in culture, together with the indirect evidence from the in vivo studies cited above, would suggest that c-fos upregulation is likely to be linked, at least in part, to increased NMDA receptor activity.

We did not comprehensively investigate c-fos expression in neuropathic pain models, since published results on c-fos expression in neuropathic pain models, particularly in the chronic phase, have been extremely inconsistent, even in the rat, which has been studied fairly extensively [64]. However, instead, we did examine one of the potential cellular mechanism, through which EphB1 deficiency may modulate neuropathic pain, based on a growing body of evidence implicating microglial activation in neuropathic pain, which would lead to pro-inflammatory responses with pathological effects [28], [65]. We therefore counted activated microglia in the dorsal horn of the spinal cord of WT and EphB1 KO mice following PNL. Possibly due to the fact that many of the studies on the molecular mechanism regulating microglial activation have been conducted in vitro, the precise nature of the cellular and molecular mechanisms regulating this activation in vivo is still poorly understood, but the current consensus is that activation is triggered by signals coming from primary afferents, with prominent candidates being ATP and monocyte chemoattractant protein-1 (MCP-1), followed by neuronal factalkine, cleaved by Cathepsin S of microglial origin [28], [66], Furthermore, input from A fibres has been shown to be important for microglial activation in the spared nerve model [67]. None of these mechanisms should be affected by lack of EphB1 receptors. However, microglial activation was attenuated in EphB1 KO mice from day 3, in parallel with the observed attenuation of thermal and mechanical hyperalgesia. This would suggest that the EphB1 receptor is implicated in the mechanisms leading to microglia activation in neuropathic pain, even though other mechanisms, independent of EphB1 receptor expression, must be involved, since some level of microglial activation was present also in KO mice. We had observed an analogous reduction in microglial activation in a conditional knockout (generated in a different mouse strain), lacking ephrinB2 specifically in a subpopulation (largely nociceptive) of DRG neurons [49], confirming that the observations made here in EphB1 KO mice cannot be due to the lack of presumptive pre-synaptic EphB1 receptors. This would indicate that microglial activation must be at least in part mediated by post-synaptic mechanisms. Daulhac et al. [68] observed, in a model of diabetic neuropathy, that microglia activation was dependent on NMDA receptor activity. The findings presented here would suggest that a similar mechanism may also be at work in our injury model of neuropathic pain, since lack of EphB1 receptors can affect NMDA receptor function. Further studies should be carried out on these potential post-synaptic mechanisms, also to determine if they contribute to microglial activation in all types of pain. In contrast to Dauhlac et al. findings in diabetic neuropathy, Cheng et al, [69] observed NMDA-independent microglia activation in CFA-induced inflammatory pain.

Interestingly, both thermal and mechanical hyperalgesia are still present at day 21 in WT, and to a lesser extent in EphB1 KO mice, when microglial activation is no longer detectable, supporting the hypothesis that microglial activation is important for the early phases of neuropathic pain, rather than long-term maintenance.

Our findings can be explained in their entirety by postulating an exclusively post-synaptic site of action for EphB1 receptors. Nevertheless two of the studies by Song’s group [9], [25] would suggest that, at least in the CCI model, there may be an involvement of pre-synaptic EphB1 receptors in the behavioural responses observed. They demonstrated hyperexcitability of DRG neurons following CCI, which was not present in EphB1 KO mice and which could be blocked by EphB1-Fc. Also in rats CCI led to increased expression of EphB1 in DRG neurons. Kobayashi et al. [24] suggested the presence of ephrin B2 in spinal cord neurons, and this could potentially activate EphB1 receptors on DRG neurons. Any pre-synaptic role of EphB1 receptors is however likely to be in addition to, rather than in alternative, to a post-synaptic role, since both EphB1 and NMDA receptors have been shown to be present in dorsal horn neurons, and can be immunoprecipitated together from the spinal cord. Crucially, in a nociceptor-specific ephrinB2 deficient mouse we showed a reduced phosphorylation of NR2B in the formalin and PNL models [49]. This reduction could not be mediated by pre-synaptic EphB1 receptors, since their potential ephrinB2 ligands in the spinal cord are still present in the ephrin B2 nociceptive specific KO. Also, the findings we reported with ephrin B2 nociceptive specific KO mice allow us to exclude that the results we report here may be due to EphB1 receptors in the dorsal horn acting by activating reverse signalling via pre-synaptic ephrinB2; as mentioned in the introduction, EphB receptor-ephrinB interactions may activate both forward signalling via the receptor, and reverse signalling via the transmembrane ligand, and there is evidence, mainly in the hippocampus, that ephrin signalling may modulate synaptic function [6].

Moreover, it is not clear whether any possible role played by EphB1 receptors in DRG neurons would be at the level of central synapses, or rather in the periphery, where at least in the case of tissue damage models (formalin), peripheral EphB receptors appear to be important [70]. It is however worth noticing that the role identified by Cao et al. [70] for EphB receptors in the periphery may not be mediated entirely (or at all) by EphB1 receptors, but by another EphB receptor. Intraplantar injection of EphB1-Fc (which would block any EphB receptor) reduced pain-related behaviour in both phase I and phase II in Cao et al.’s experiments, whereas in EphB1 KO mice the response in Phase I is not affected. A peripheral site of action for EphB1 receptors on DRG neurons could still explain the differences in excitability observed by Han et al. [25] in injured EphB1 KO mice.

### Conclusions

Independently of the differences we observed in the extent of the effect of the lack of functional EphB1 receptors in a variety of inflammatory, tissue and nerve injury models, in all cases we found that the lack of EphB1 protein led to a blunted or absent thermal and mechanical hyperalgesia, or spontaneous pain behaviour. It would therefore appear likely that the induction and/or maintenance of central sensitisation require the presence of EphB1 receptors, regardless of cause and sensory modality examined. Cellular and molecular mechanisms that mediate the role of EphB1 in leading to increased sensitivity/spontaneous pain are likely to include NR2B phosphorylation,increased c-fos expression and microglial activation. Our present findings confirm the identity of the EphB receptors involved in the onset of various types of persistent, chronic pain as the EphB1 receptors, extend the involvement of EphB receptors to models of long term inflammation and the Seltzer neuropathic pain model, suggesting that this receptor is probably involved in all forms of allegedly NMDA-dependent persistent pain. Intriguingly, in models of persistent pain we observed that EphB1 KO mice developed hyperalgesia/allodynia, but recovered faster as compared to WT mice, suggesting that the EphB1 receptor may be an attractive target for the development of analgesic strategies.
